# Robot-Controlled Acupuncture—An Innovative Step towards Modernization of the Ancient Traditional Medical Treatment Method

**DOI:** 10.3390/medicines6030087

**Published:** 2019-08-10

**Authors:** Kun-Chan Lan, Gerhard Litscher

**Affiliations:** 1 Department of Computer Science and Information Engineering, National Cheng Kung University, Tainan 704, Taiwan; 2 Traditional Chinese Medicine (TCM) Research Center Graz, Research Unit of Biomedical Engineering in Anesthesia and Intensive Care Medicine, and Research Unit for Complementary and Integrative Laser Medicine, Medical University of Graz, 8036 Graz, Austria

**Keywords:** robot-controlled acupuncture (RCA), robot-assisted acupuncture (RAA), computer-controlled acupuncture (CCA), high-tech acupuncture

## Abstract

**Background:** For several years now, research teams worldwide have been conducting high-tech research on the development of acupuncture robots. In this article, the design of such an acupuncture robot is presented. **Methods:** Robot-controlled acupuncture (RCA) equipment consists of three components: (a) Acupuncture point localization, (b) acupuncture point stimulation through a robotic arm, and (c) automated detection of a deqi event for the efficacy of acupuncture point stimulation. **Results:** This system is under development at the Department of Computer Science and Information Engineering, National Cheng Kung University, Tainan. Acupuncture point localization and acupuncture point stimulation through a robotic arm works well; however, automated detection of a deqi sensation is still under development. **Conclusions:** RCA has become a reality and is no longer a distant vision.

## 1. Introduction

Robots are an indispensable part of healthcare today. They perform services silently and in the background, transporting medical equipment and material or laundry, and shaking test tubes or filling in samples. However, should a robot perform an operation or can it make a diagnosis? Most of us will have strong reservations on this. We all want an experienced person to conduct operations. The use of robots in the operating room, however, does not exclude the experienced physician; on the contrary, robots are ideal at supporting them at work. A robot cannot and should not replace a doctor, but can be an important asset. Like other tools, it can have a variety of shapes. For example, medical surgical systems can be more flexible than a human and can have more than two arms that are moved by a console controlled by the physician. Thus, the various robots in medicine are special tools that can support the services of the doctor and ensure better results. The robot’s role as a team member in the operating room will certainly be enhanced in the future. The fact that the robot with its high degree of specialization can help people and work tirelessly makes medicine safer. So why waive the support of such a helper in acupuncture and not benefit from its many advantages?

**Figure 1:**
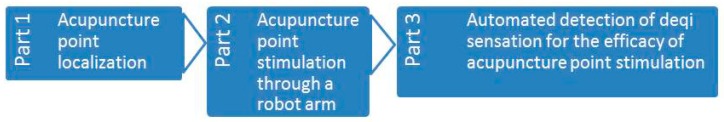
Schematic presentation of the robot-controlled acupuncture system. Note: Part 3 is still under development.

In this article, the design of an automated robotic acupuncture system is presented. The system consists of three main components: (1) Acupuncture point localization, (2) acupuncture point stimulation through a robotic arm, and (3) automated detection of a possible deqi event for the efficacy of acupuncture point stimulation (developmental stage). The overall system design is schematically shown in Figure 1.

Parts 1 and 2 of the system were developed at the Department of Computer Science and Information Engineering at the National Cheng Kung University in Tainan. The first measurements will take place at the Traditional Chinese Medicine (TCM) Research Center, Medical University of Graz.

## 2. Acupuncture Point Localization 

TCM is a system that seeks to prevent, diagnose and cure illnesses, dating back to several thousand of years. In TCM, qi is the vital energy circulating through channels called meridians, which connect various parts of the body. Acupuncture points (or acupoints) are special locations on meridians, and diseases can be treated or relieved by stimulating these points. However, it is not easy to remember all of the acupoint locations and their corresponding therapeutic effects without extensive training.

Traditionally, people use a map or dummy to learn about the locations of acupuncture points. Given that now over one-third of the world’s population owns a smartphone [1], a system using Augmented Reality (AR) is proposed to localize the acupuncture points. The proposed system includes three stages: Symptom input, database search and acupuncture point visualization.

(1) Symptom input: The user interacts with a chatbot on the smartphone to describe her/his symptoms.
(2) Database search: According to the symptom described by the user, a TCM database is searched and symptom-related acupuncture points are retrieved.
(3) Acupoint visualization: Symptom-related acupoints are visualized in an image of the human body.

Compared to the traditional acupoint probe devices that work by measuring skin impedance, the presented system does not require any additional hardware and is purely software-based. In the case of mild symptoms (e.g., headache, sleep disorder), with the aid of the proposed system, the patient can quickly locate the corresponding acupuncture points for the application of acupuncture or massage, and relieve her/his symptoms without special help from TCM physicians.

While there are studies [2,3] that propose similar ideas (the use of AR for acupoint localization), they did not consider the effect of different body shapes and changes of the viewing angle on the accuracy of acupoint localization. In this project, several techniques, including facial landmark points, image deformation, and 3D morphable model (3DMM) are combined to resolve these issues. Due to clarity, the focus of the discussion on localizing acupoints should be on the face, because it is one of the most challenging parts of the human body, with regards to RCA.

### 2.1. Image-Based Acupoint Localization 

Jiang et al. proposed Acu Glass [2] for acupoint localization and visualization. They created a reference acupoint model for the frontal face, and the acupoint coordinates are expressed as a ratio to face bounding box (returned by the face detector). The reference acupoints are rendered on top of the input face based on the height and width of the subject’s face and the distance between the eyes, relative to the reference model. They used Oriented FAST and Rotated BRIEF (ORB) feature descriptors to match feature points from the current frame and the reference frame, to obtain the estimated transformation matrix. Instead of scaling the reference acupoints like [2], Chang et al. [3] implemented a localization system based on cun measurement. Cun is a traditional Chinese unit of length (its traditional measure is the width of a person’s thumb at the knuckle, whereas the width of the two forefingers denotes 1.5 cun and the width of four fingers side-by-side is three cuns). They used the relative distance between landmarks to convert pixel into cun, assuming that the distance between hairline and eyebrow is 3 cun. The acupoint can be located based on its relative distance (in cun) from some landmark point. However, there are issues with the above approaches for acupoint localization, including:

(i) The differences of face shape between different people are not considered
(ii) A conversion of pixel to cun, based on the frontal face, cannot be directly applied to locating acupoints on the side of the face.

Since landmark points contain shape information, all landmark points should be considered, instead of just a few being used to estimate the location of an acupoint (like in [3]). In the current project, landmark points are used as the control points for image deformation, and the locations of acupoints can be estimated by deforming a reference acupuncture model. In addition, the reference acupuncture model is allowed to be changed dynamically to match different viewing angles. More specifically, a 3D model with labeled acupoints is created so that the 3D model can rotate to match the current viewing angle. Finally, a 3D morphable model for different face shapes is provided. In other words, a 3D morphable model is deformed to best fit the input face, and the acupoints are projected to 2D as the reference acupuncture model. A summary of previous studies in acupoint localization is shown in Table 1.

### 2.2. Landmark Detection

A marker is often used in AR for localization. Instead of putting markers on the user, landmark points and landmark detection are often used for markerless AR. The processing overhead of the landmark detection algorithm is vital for our application due to its real-time requirement. Therefore, the complexity of the algorithm must be considered. Real-time face alignment algorithms have been developed [4,5], and the Dlib [6] implementation by [4] is used in the present work.

### 2.3. Image Deformation

Image deformation transforms one image to another, wherein the transformed image is deformed from the original image by being rotated, scaled, sheared or translated. A scaling factor can be estimated with two sets of corresponding points. Jiang et al. [2] used the distance between eyes and the height of the face as the scaling factor of the x and y directions. Then the same transformation is applied to every pixel on the reference acupoint model. However, given that their scaling factors are based on a frontal face model, it cannot be applied to the side face. In addition, they did not consider difference face shapes. In the present work, we use a weighted transformation based on moving least squares [7]. The distance between the control points is used as the weight during the transformation. Here, the control points are the landmark points.

### 2.4. 3D Morphable Model (3DMM)

Given that the acupoints are defined in a 3D space, a 3D face model is useful for acupoint localization. The 3D Morphable Models (3DMM) [8,9,10,11] are common for face analysis because the intrinsic properties of 3D faces provide a representation that is immune to intra-personal variations. Given a single facial input image, a 3DMM can recover 3D face via a fitting process. More specifically, a large dataset of 3D face scans is needed to create the morphable face model, which is basically a multidimensional 3D morphing function based on the linear combination of these 3D face scans. By computing the average face and the main modes of variation in the dataset, a parametric face model is created that is able to generate almost any face.

After the 3DMM is created, a fitting process is required to reconstruct the corresponding 3D face from a new image. Such a fitting process seeks to minimize the difference between an input new face and its reconstruction by the face model function. Most fitting algorithms for 3DMM require several minutes to fit a single image. Recently, Huber et al. [12] proposed a fitting method using local image feature that leverages cascaded regression, which is being widely used in pose estimation and 2D face alignment. Paysan et al. [13] published the Basel Face Model (BFM), which can be obtained under a license agreement. However, they did not provide the fitting framework. On the other hand, Huber et al. [14] released the Surry Face Model (SFM), which is built from 169 radically diverse scans. SFM is used in the present work, as it provides both the face model and the fitting framework.

### 2.5. Face Detection (FD) and Tracking

Haar-like features [15] with Adaboost learning algorithm [16,17] have been commonly used for object detection. However, they tend to have a high false positive rate. Zondag et al. [18] showed that the histogram-of-oriented-gradient (HOG) [19] feature consistently achieves lower average false positive rates than the Haar feature for face detection. In the present work, a face detector based on HOG-based features and support vector machine (SVM) were implemented. However, the computation overhead to combine HOG and SVM could be a bit significant when running the face detector on a smartphone. In addition, a low detection rate might occur for a fast-moving subject. For example, during the process of acupuncture or acupressure, some part of the face might be obscured by the hand such that the face is undetected by the face detector. Therefore, a face-tracking mechanism has been implemented to mitigate these problems.

A correlation-based tracker is known for its fast tracking speed, which is vital for the application. Bolme et al. proposed the minimum output sum of squared error (MOSSE) based on the correlation filter [20]. Danelljan et al. introduced the discriminative scale space tracker (DSST) [21] based on the MOSSE tracker. DSST can estimate the target size and multi-dimension feature such as PCA-HOG [22], which makes the tracker more robust to intensity changes. The Dlib [6] implementation of DSST [21] is used in this work.

### 2.6. Method—Acupuncture Point Localization

**Figure 2:**
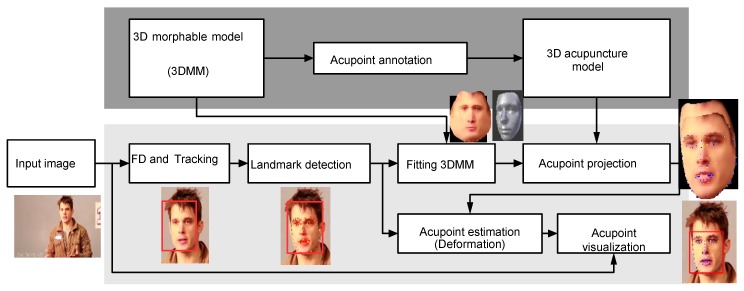
Flow chart of 3D acupuncture point estimation.

A flow chart of the 3D acupuncture point localization is shown in Figure 2. The offline process is shown in dark gray and online process in light gray. The acupoints are labeled in the offline process. During the online process, face detection is performed on the input image, and then landmark detection is applied to the face region to extract facial landmark points.

The landmark points are then utilized in the fitting process of 3D morphable model (3DMM) to have a fitted model to the input face, and the 3D model is then rotated to the same angle as the input face. Next, landmark points on the 3D model will be projected onto 2D. During the acupoint estimation process, the projected acupoints are deformed using the control points, which contain the projected landmark points and the input landmarks points. Finally, the estimated acupoints are warped into the input image to visualize the acupoints.

#### 2.6.1. 3D Morphable Model

A facial 3D morphable model (3DMM) is a static model generated from multiple 3D scans of human faces. The 3DMM can be used to reconstruct a 3D model from a 2D image. In this work, Surry Face Model (SFM) [14] is used to implement our 3DMM model. The generated model is fitted using the landmark points on the 2D image, such that different face shapes can be considered.

The generated 3D model is expressed as S = [x_1_, y_1_, z_1_, . . . , x_n_, y_n_, z_n_]^T^, where [x_n_, y_n_, z_n_]^T^ is the n-th vertex. Principal component analysis (PCA) is applied to the 3DMM, which yields a number of m components. A 3D model can be approximated by linear combination of the basis vectors S = v¯+ ∑^m^ α_i_v_i_, where v¯ is mean mesh, α is the shape coefficients, v is the basis vector, m is the number of principal components.

#### 2.6.2. Acupoint Annotation

**Figure 3:**
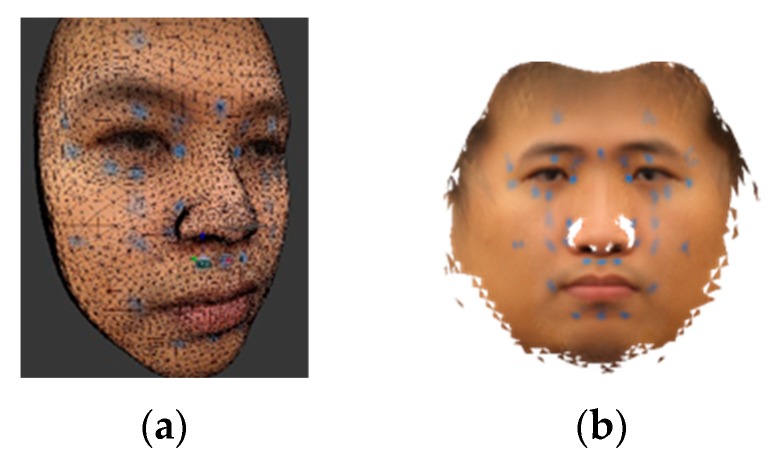
3D acupoint annotation. (**a**) acupoint annotation, (**b**) isomap texture.

Face images from a large number of subjects with different face angels should be captured, and blue stickers put on their faces to annotate the acupoints. To define an acupoint reference model, a mean face for thirteen different face angles will be built, and all mean faces then merged into an isomap texture, as shown in Figure 3b. The texture and mean model of the 3DMM are then loaded into the 3D graphics software. For every acupoint, the 3D coordinates of the center point of the blue sticker and its three nearest vertices are marked. The coordinates of the landmark points are marked as well.

#### 2.6.3. 3D Acupuncture Model

The 3D acupuncture model contains labeled metadata during the step of acupuncture annotation, including the indices of landmark vertex (denoted as Lindex), the 3D coordinates of acupoints (denoted as Aref 3D), and the indices of three nearest vertices to the acupoint (denoted as AneiIndex).

#### 2.6.4. Face Detection and Tracking

We plan to implement a face detector using SVM with HOG-based features. In addition, a DSST-based tracker will be employed to speed up the system and reduce face detection failure when the face is occluded by the hand during acupuncture or acupressure. The face is tracked by the tracker once the face is detected. 

Peak to Sidelobe Ratio (PSR) [23] is a metric that measures the peak sharpness of the correlation plane, commonly used to evaluate tracking quality. For estimation of the PSR, the peak is located first. Then the mean and standard deviation of the sidelobe region, excluding a central mask centered at the peak, are computed. In this work, a 11x11 pixel area is used as the central mask. 

Once the face is detected, the face area is used to calculate filter Hl, which is then used to track the face for future frames. If the PSR is lower than a defined threshold, the face is re-detected to reduce the tracking loss. For every FD frame, we select a region around the current face image. The selected region is slightly larger than the face region and we try to re-locate the face region in order to keep the region constrained. The constrained face region improves the accuracy of the landmark detection.

#### 2.6.5. Landmark Detection

For concerns of computation speed, an ensemble of regression trees is used in our work for landmark detection [4]. The difference of pixel intensity between a pair of landmark points are selected as the feature used to train the regression tree. Each tree-based regressor is learned using the gradient boosting tree algorithm [24].

#### 2.6.6. Fitting 3DMM

2D landmarks are used in the process of fitting 3DMM to find optimal PCA coefficients. The goal is to minimize the re-projected distance of the landmark points.

#### 2.6.7. Acupoint Projection

Pose estimation problem is often referred as perspective-n-point (PNP) problem [25,26]. Given the input of a set of 2D points and its corresponding 3D points, a pose estimation function estimates the pose by minimizing re-projection errors. The output of such a function contains information that can transform a 3D model from the model space to the camera space. We plan to utilize the pose estimation algorithm included in the SFM fitting framework [14]. In our case, the 2D points set are obtained from landmark detection, while the 3D points are labeled at the offline stage. 

#### 2.6.8. Acupoint Estimation

The acupoint estimation problem is treated as an image deformation process, in which image deformation using moving least square [7] is used. The basic image deformation affine transform is defined as l_v_(x) = Mx + T, where M is a matrix controls scaling, rotation and shear, x is a 2D point, T controls the translation. Let f be a function of image deformation, f(p) = q transform a set of control points p to a new position q.

**Figure 4:**
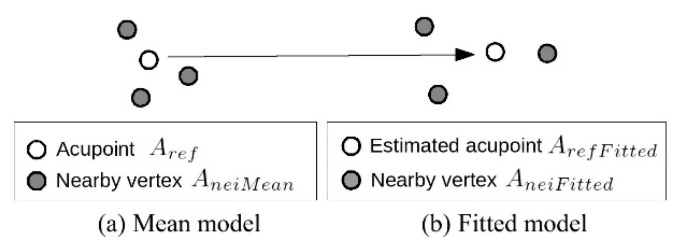
Visualized acupoint during the estimation process. (**a**) Mean model, (**b**) Fitted model.

As shown in Figure 4, since the reference acupoints A_ref_ are only available in the mean model, the actual acupoint location A_refFitted_ in the fitted model will be estimated using the nearby vertices as the control points (see Equations (1) and (2)).

A_refFitted_ = deform(A_neiMean_, A_neiFitted_, A_ref_) (1)

A_in_ = deform(L_fitted_, L_in_, _Aref F itted_) (2)

#### 2.6.9. Acupoint Visualization

**Figure 5:**
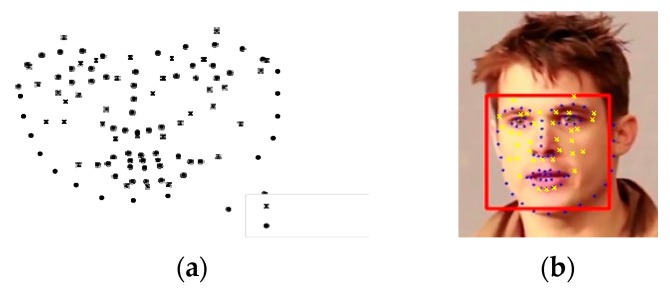
Acupoint visualization. (**a**) acupuncture model; (**b**) warped acupoints.

Landmark points (denoted as dots) and acupoints (denoted as crosses) are illustrated in Figure 5a. The landmark points in the input image are used as the destination control points, while landmark points in the reference model (i.e. the fitted 3DMM model) are used as the control points to deform the reference acupoints. The deformed acupoints are then visualized onto the input image, as shown in Figure 5b. The warping not only visualizes the acupoints, but also takes care of the non-rigid part of the face, such as facial expressions, the mouth opening, etc.

## 3. Automated Acupoint Stimulation

**Figure 6:**
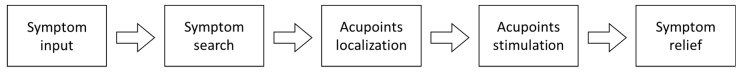
System architecture.

The second part of this future-oriented work is to build a system for automated acupuncture. Given that a depth camera is still quite expensive (8–10 times more expensive than a 2D camera), this project plans to build an automated acupuncture system with an inexpensive 2D monocular camera and a robot arm. The main research issue is identifying how to localize the acupoint from the image space to the robot arm space. The system architecture is shown in Figure 6.

### 3.1. Hand-Eye Calibration

The problem of determining the relationship between the camera and the robot arm is referred to as the hand-eye calibration problem, which is basically to transform the target coordinate position in the image plane to the corresponding coordinate position in the robot arm plane. Due to the use of a 2D camera, we limit the conversion from the 2D image plane to the robot arm X-Y plane. In this project, perspective coordinate transformation is used, also called 8-parameter coordinate transformation [27], which does not have to maintain parallelism compared to other coordinate transformation methods. 

**Figure 7:**
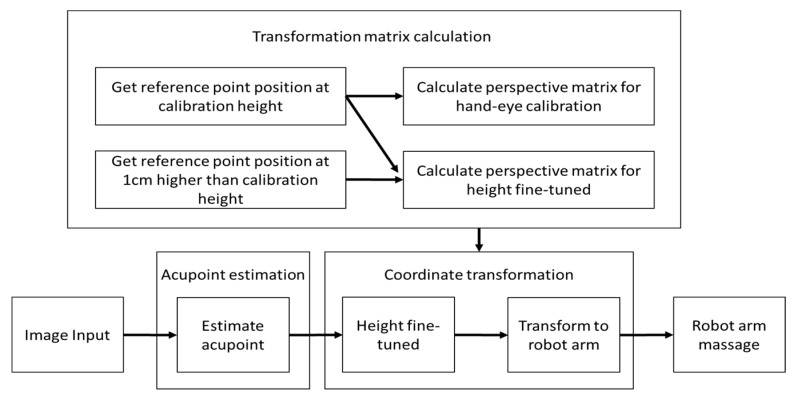
System components for robot arm acupuncture or massage.

The present system can be divided into three big parts—acupoint estimation, transformation matrix calculation and coordinate transformation. Transformation matrix calculation estimates the matrix used by coordinate transformation and only performs at the first time of setting up the system (Figure 7). The camera first reads an image as input, and then moves to the acupoint estimation part (as discussed in the previous acupoint localization section) to get the image coordinates of the acupuncture points. Then, the acupoint image coordinate is passed to the coordinate transformation part to get the robot arm coordinates of the acupoints. 

### 3.2. Transformation Matrix Calculation

Perspective transformation transform coordinates between image plane and robot arm plane was used here:

(3) $$X=\frac{ax+by+c}{gx+hy+1}\;Y=\frac{dx+ey+f}{gx+hy+1}$$

Perspective equation (3) shows that one can transform the coordinate from the original coordinate (x,y) to the destination coordinate (X,Y). The eight parameters (a–h) control in-plane-rotation, out-plane-rotation, scaling, shearing, and translation. The goal is to calculate the eight unknowns (a–h), so that Equation (5) can be used to transform coordinates from origin to destination. With these eight unknowns (a–h), at least four known points in both systems are required. Intuitively, using more known reference positions to solve the equation can reduce errors and increase accuracy. If we have more than four known points in both systems to solve eight parameters’ transformation, we call this as an overdetermined system. The method of least squares is a standard approach in regression analysis to approximate the solution of overdetermined systems. Considering the time and distribution, we selected 13 reference points to obtain 13 known points in both systems and solve the perspective transformation equation. We can simply find the least squares solution by using linear algebra (4):

(4) $$Ax=b\Rightarrow x={\left(A^{T}A\right)}^{-1}A^{T}b$$

(5) X=ax+by+c−gxX−hyX    Y=dx+ey+f−gxY−hyY 

Since the original equation is nonlinear (3), we have to linearize (5) it in order to solve it using linear algebra.

**Figure 8:**
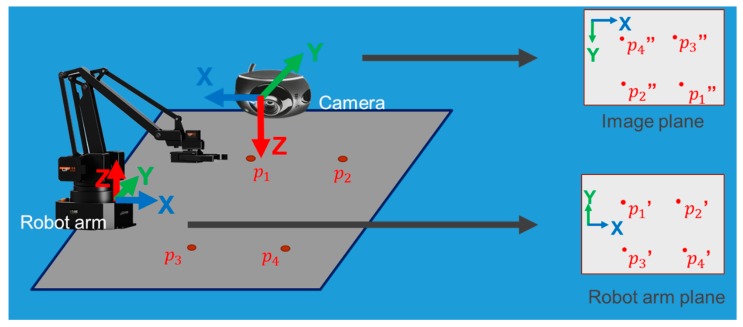
Diagram of hand-eye calibration.

Finally, it can be converted to Ax=b, and Equation (4) is used to find the least squares solution of the perspective transformation. We apply perspective transformation to the camera and robot arm to conduct hand-eye calibration, as shown in Figure 8. We take the image plane as the original coordinate and the robot arm plane as the destination coordinate, in order to calculate perspective matrix T (6,7).

(6) $$\left|\begin{array}{cccccccc} x_{1} & y_{1} & 1 & 0 & 0 & 0 & -x_{1}X_{1} & -y_{1}X_{1}\\ x_{2} & y_{2} & 1 & 0 & 0 & 0 & -x_{2}X_{2} & -y_{2}X_{2}\\ x_{3} & y_{3} & 1 & 0 & 0 & 0 & -x_{3}X_{3} & -y_{3}X_{3}\\ x_{4} & y_{4} & 1 & 0 & 0 & 0 & -x_{4}X_{4} & -y_{4}X_{4}\\ 0 & 0 & 0 & x_{1} & y_{1} & 1 & -x_{1}Y_{1} & -y_{1}Y_{1}\\ 0 & 0 & 0 & x_{2} & y_{2} & 1 & -x_{2}Y_{2} & -y_{2}Y_{2}\\ 0 & 0 & 0 & x_{3} & y_{3} & 1 & -x_{3}Y_{3} & -y_{3}Y_{3}\\ 0 & 0 & 0 & x_{4} & y_{4} & 1 & -x_{4}Y_{4} & -y_{4}Y_{4} \end{array}|\cdot |\begin{array}{c} a\\ b\\ c\\ d\\ e\\ f\\ g\\ h \end{array}|=|\begin{array}{c} X_{1}\\ X_{2}\\ X_{3}\\ X_{4}\\ Y_{1}\\ Y_{2}\\ Y_{3}\\ Y_{4} \end{array}\right|$$

(7) $$P^{\prime }=T*P^{\prime \prime }\;P^{\prime \prime }=\left\{p_{1}^{\prime \prime },p_{2}^{\prime \prime },p_{3}^{\prime \prime },p_{4}^{\prime \prime },\ldots \right\}\;P^{\prime }=\left\{p_{1}^{\prime },p_{2}^{\prime },p_{3}^{\prime },p_{4}^{\prime },\ldots \right\}\;T:perspective\;matrix\;from\;image\;plane\;to\;robot\;arm\;plane$$

**Figure 9:**
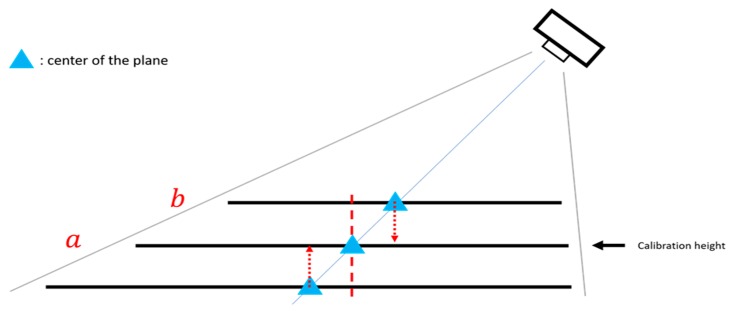
Errors caused by different heights.

It is to be kept in mind that the camera might be placed at the side of the image and different acupoints might have different heights; therefore, when we project the coordinates of the actual height of the acupoint to the calibration height, it might not be the point of the acupoint position we see in the image, which could result in an estimation error, as shown in Figure 9.

**Figure 10:**
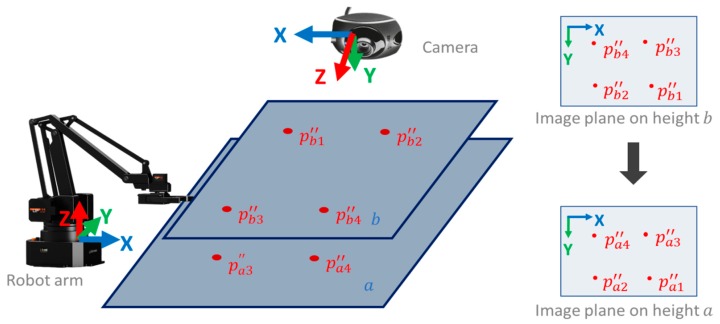
Diagram of the fine-tuned height.

Therefore, perspective transformation is again applied to the image plane at a different height to obtain the fine-tuned height matrix as shown in Figure 10. The image plane is taken at the calibration plane height (plane a) as the original coordinate and the image plane at the height higher than the calibration height (plane b) as the destination coordinate to calculate perspective matrix F, as shown in Figure 10.

**Figure 11:**
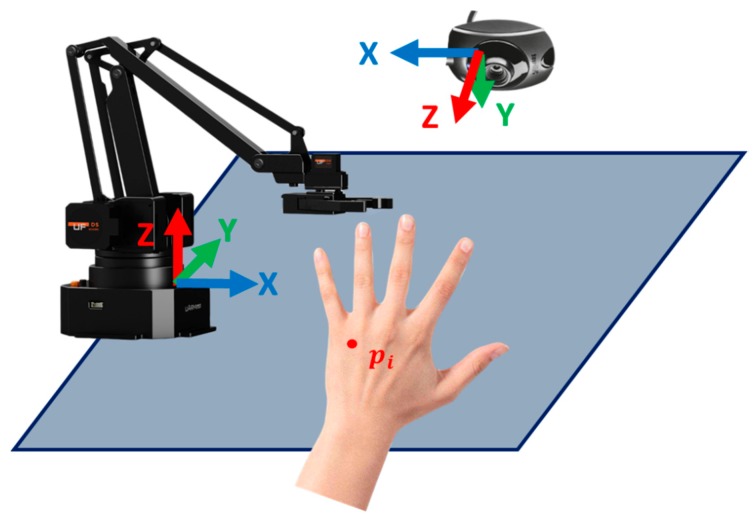
Diagram of the practical application.

Combining Equations (7) and (8), we get Equation (9), which converts the acupoint from the image space to the robot arm space, as shown in Figure 11.

(8) $$P_{a}^{\prime \prime }=F*P_{b}^{\prime \prime }\;P_{b}^{\prime \prime }=\left\{p_{b1}^{\prime \prime },p_{b2}^{\prime \prime },p_{b3}^{\prime \prime },p_{b4}^{\prime \prime },\ldots \right\}\;P_{a}^{\prime \prime }=\left\{p_{a1}^{\prime \prime },p_{a2}^{\prime \prime },p_{a3}^{\prime \prime },p_{a4}^{\prime \prime },\ldots \right\}\;a:\mathrm{calibration}\;\mathrm{plane}\;\mathrm{height}\;b:\mathrm{heigher}\;\mathrm{than}\;\mathrm{calibration}\;\mathrm{plane}\;\mathrm{height}\;F:perspective\;matrix\;from\;image\;plane\;on\;height\;b\;to\;\;image\;plane\;on\;height\;a$$

(9) $$p_{i}^{\prime }=T(p_{i}^{\prime \prime }+\left(\frac{k}{b-a}\right)\left(Fp_{i}^{\prime \prime }-p_{i}^{\prime \prime }\right))\;p_{i}^{\prime \prime }:acupoint\;image\;coordinate\;p_{i}^{\prime }:tip\;of\;robot\;arm\;coordinate\;k:height\;of\;the\;acupoint$$

## 4. Automated Detection of Deqi Event

The arrival of qi (deqi) is considered to be related to acupuncture efficacy [28]. Deqi can be measured by the sensation of the acupuncturists and the reaction of the patient.

While a deqi sensation scale is considered an important qualitative and quantitative measuring tool, there is no standardization or reliable deqi scale due to lack of sufficient evidence [29,30,31,32]. In 1989, the Vincent deqi scale was invented based on the McGill pain questionnaire. The Park deqi scale and MacPherson deqi scale followed [33,34].

The Massachusetts General Hospital acupuncture sensation scale (MASS) [35] is composed of various needling sensations and has a system that measures the spread of deqi and patient’s anxiety levels during needling. These scales mainly focus on the patient’s sensations and are pretty subjective. Ren et al. [36] conducted a survey to determine the perspectives of acupuncturists on deqi. Again, their results are based on subjective questionnaires collected from a small number of acupuncturists [36].

The effects of acupuncture are well reflected in electroencephalogram (EEG) changes, as revealed by recent reports [37,38,39]. Therefore, the EEG can also be used as an objective indicator of deqi, as its change is significantly associated with autonomic nervous functions. Observing EEG changes in relation to acupuncture is more objective than using a questionnaire.

While observing the deqi effect via EEG is considered a more subjective measure, the results from prior studies are not consistent. Yin et al. [40] observed significant changes in alpha band electroencephalogram powers during deqi periods, while Streitberger et al. [41] only found significant changes in alpha1 band. Other studies [37,39,42,43] have shown that EEG power spectrum increases in all frequency bands, including alpha-wave (8–13 Hz), beta-wave (13–30 Hz), theta-wave (4–8 Hz), and delta-wave (0.5–4 Hz) in the frontal, temporal, and occipital lobes when a deqi effect is reported by the subjects. Note that the number of subjects in these studies are generally small (10–20 people) and different acupuncture points are used in different studies. Furthermore, while deqi sensation is commonly believed to be the most important factor in clinical efficacy during acupuncture treatment, the detection of deqi mostly relies on the participant’s subjective sensations and sometimes the acupuncturist’s perceptions. It has been found that there can be some differences between a patient’s real-life experience and the acupuncturist’s sensation [44]. In clinical practice, many acupuncturists can only detect deqi effects based on the patient’s report or observation of the reaction of a patient to deqi.

In this context, there is the plan to design a portable device based on collection of EEG signals to automatically detect a deqi effect. Such a device can be useful for clinical practice and acupuncture training in schools. It should be developed such that it automatically detects deqi events and reports it wirelessly to the acupuncturist.

## 5. Discussion

Reports on medical robotics [45] show applications in the field of acupuncture. In the humor section for the online publication, Science-based Medicine, Jones [46] picturizes a patient being treated for fatigue syndrome by an acupuncturist and an acupuncture anesthetist, who use robotic acupuncture. 

It is interesting, as Jones states, that one of the greatest strengths of acupuncture, namely the direct connection between the doctor and the acupuncture needle, is also one of its major weaknesses. As mentioned in the introduction, surgeons use robotic technology to perform an increasing number of minimally invasive procedures [45].

The healing art of acupuncture is deeply rooted in ancient Eastern culture, but the modern technology that is now being used to expand it comes from Western medicine [45,46]. Jones says that state-of-the-art medical robotic technologies feature high-resolution 3D magnification systems and instruments that can maneuver with far-greater precision than the human wrist and fingers. This allows specially-trained acupuncturists to locate and successfully control hard-to-reach acupuncture points (those next to the eye or in the genital area). This could expand the number of indications suitable for acupuncture [45].

However, robot-assisted acupuncture is not the only high-tech therapeutic option in the field of complementary medicine. Other methods have been incorporating modern technology into their protocols for years. Chiropractors, for example, adopt the latest high-tech electronics devices. This helps facilitate the localization of subluxations in the spine. Once a safe diagnosis is made, it can be treated using traditional hands-on techniques [45,46].

## 6. Conclusions

In 1997, our Graz research team at the Medical University of Graz in 1997 showed how acupuncture worked without the aid of an acupuncturist and we introduced the term “computer-controlled acupuncture” [47]. However, we did not imply that the computer would replace the acupuncturist; rather, we were seeking to highlight the quantification of the measurable effects of acupuncture. This vision of “robot-controlled acupuncture” is now a reality [47,48,49,50]; the research work in this article clearly shows its present status. Further modernization of acupuncture, like automatic artificially-based detection of dynamic pulse reactions for robot-controlled ear acupuncture, is under development.

## Figures and Tables

**Table 1:** Comparison of related work.

Method	Angle-aware	Shape-aware	Reference Model	Estimation	Limitation
Chang et al. [2]	No	No	2D	Using cun measurement system by first converting pixel into cun	1) Assuming that the hairline is not covered with hair 2) Does not work for side face
Jiang et al. [3]	Partially	No	2D	Scaling of the reference model	1) The scaling factor is based on the bounding box ratio returned by the edge detector, which can be unreliable 2) Does not work for side face
Proposed system	Yes	Yes	3D	3DMM, weighted deformation, landmarks	Need landmark points
